# Serum Natrium Determines Outcome of Treatment of Advanced GIST with Imatinib: A Retrospective Study of 80 Patients from a Single Institution

**DOI:** 10.5402/2011/523915

**Published:** 2011-09-07

**Authors:** Ninna Aggerholm-Pedersen, Peter Rasmussen, Helle Dybdahl, Philip Rossen, Ole Steen Nielsen, Akmal Safwat

**Affiliations:** ^1^Department of Oncology, Aarhus University Hospital, Nørrebrogade 44, 8000 Aarhus C, Denmark; ^2^Department of Surgery, Aarhus University Hospital, Nørrebrogade 44, 8000 Aarhus C, Denmark; ^3^Department of Pathology, Aarhus University Hospital, Nørrebrogade 44, 8000 Aarhus C, Denmark; ^4^Oncology Department, College of Medicine, King Saud University, 11411 Riyadh, Saudi Arabia

## Abstract

Treatment with tyrosine kinase inhibitors (TKIs) has drastically improved overall survival (OS) of patients with advanced GIST. The aim of this study is to evaluate the results of treatment with different TKIs on advanced GIST and identify prognostic factors for OS. The medical records of all patients treated at the Department of Oncology, Aarhus University Hospital were retrospectively reviewed. Between 2001 and 2009, 80 patients with advanced GIST were treated with imatinib as first-line therapy. The median OS was 44 months (95% CI 31–56), and the 5-year OS was 40%. Since 2005, 32 patients were treated with sunitinib as 2nd-line therapy. The median time to progression was 9 months (95% CI: 3–13 months), and the 3-year OS was 30%. The data illustrate that data from large multicenter studies are reproducible in a single sarcoma centre. This retrospective study pointed to low serum sodium at the start of imatinib as a possible prognostic factor affecting OS.

## 1. Introduction

Gastrointestinal stromal tumour (GIST) is an uncommon malignancy with an estimated annual incidence of 14/million meaning that 50–60 new cases are diagnosed in Denmark each year. GIST often occurs in the 6th and 7th decade [1]. Surgery is the standard treatment of primary GIST but not always curative as approximately 30–50% of all radically operated patients experience a relapse [2–4].

Insights into the role of Kit signal transduction in the development of GIST (a gain of function mutation in KIT and PDGEF genes) has lead to a reliable phenotypic marker for GIST, the CD117 antigen [5]. 

The KIT gene encodes a tyrosine kinase which is inhibited by targeted drugs such as imatinib. Since the approval in 2001, imatinib has become the recommended 1st-line treatment of unresectable and/or metastatic GIST, and imatinib has dramatically improved the overall survival (OS) for this group of patients [6–9]. 

Poor performance status, high neutrophil count, low haemoglobin level, male sex, low serum albumin, as well as different types of c-Kit mutations have been identified as poor prognostic factors for advanced GIST [3, 6, 8, 10, 11]. 

Data on the treatment of GIST patients with tyrosine kinase inhibitors (TKIs) are mainly gathered from large randomised clinical trials in which patients are selected according to strict inclusion criteria. Reports from individual departments on the response rates and toxicities encountered in nonselected patients in routine practice are important in order to evaluate whether data from large multicenter studies can be generalised to individual sarcoma centres. 

## 2. Patients and Methods

### 2.1. Patients

All patients with unresectable or metastatic pathologically confirmed CD117 positive GIST treated at the Department of Oncology, Aarhus University Hospital in the period 2001 to 2009 were individually evaluated. Patient data, tumour characteristic, and treatment modalities were reviewed retrospectively by systematically reviewing patient's medical records, pathology, and computerized tomography descriptions. The data were collected in a specially designed case report forms, and analyses were done using SPSS statistical package (version 18).

### 2.2. Treatment

As mutational analysis was not routinely performed, the initial dose of imatinib was left to the doctor's decision. Standard practice was imatinib mesylate (Glivec, Novartis, Switzerland) at an initial dose of 400 mg per day until progression or unacceptable toxicity was observed. In case of progression according to RECIST criteria [12], the dose of imatinib was increased to 800 mg per day until progression or unacceptable toxicity. In the year 2005, sunitinib was introduced as second-line treatment. The initial practice was to give 50 mg daily for 4 weeks followed by 2 weeks of rest. Later this was changed to a dose of 37.5 mg daily without interruption. Since January 2009 patients progressing during sunitinib were offered nilotinib 400 mg × 2 daily as 3rd-line treatment. In case of radiological verified local progression, local treatment with surgery, RFA, or stereotactic radiotherapy was performed if feasible and TKI treatment continued without modification. Clinical benefit was defined as complete response (CR), partial response (PR), and stable disease (NC) at the first evaluation after initiating the treatment. Palliative radiotherapy was used in 12% of the patients in this study and 6% received palliative chemotherapy after treatment failure with TKI treatment.

### 2.3. Medical Examination and Followup

Before start of imatinib treatment the histological diagnosis was confirmed by a specialized pathologist. The patients underwent physical examination, evaluation of performance status, haematological test, and medical history. Computerized tomography with contrast served as the method of choice for baseline and response evaluation. Response evaluation was documented according to the RECIST criteria. After the start of treatment, the patients were seen on day 14 and 28 to evaluate toxicity. If no toxicity was observed, patients were controlled every 3 months in the first 3 years and every 4 months thereafter. Toxicity grade 3 or 4 (CTC version 3) lead to dose reduction.

### 2.4. Blood Analysis

All blood samples were analysed as part of a routine laboratory procedure before start of treatment. For the statistical analysis hyponatraemia was defined as a plasma sodium level <135 mmol/L, leukocytosis as white blood cell count >10 × 10^9^/L, elevated neutrophiles as cell count >7 × 10^9^/L, and haemoglobin was defined as low when <7.4 mmol/L.

### 2.5. Statistical Analysis

The primary end points in this study were overall survival (OS) and time to progression (TTP). TTP was defined as the period from the beginning of treatment to radiological verified progression and OS from the beginning of treatment to the last day of follow-up or death (of any cause). The following potential prognostic variables were investigated for overall survival; sex, performance status (sore 0 v 1–3), concomitant diseases, and categories of the following baseline laboratory values: neutrophils, haemoglobin, lactate dehydrogenase, and sodium. For the prognostic factors evaluated each potential candidate was initially assessed by univariate analysis. Factors found to have most impact were included in a multivariate Cox regression model. For Cox multivariate model all variables were categorised for haemoglobin into high and low, performance status into good (PS0) and less (PS 1–3), and serum sodium into high and low as previously defined. A *P* value below 0.05 was considered as statistically significant. Kaplan-Meier method was used to evaluate the OS and TTP.

## 3. Results

### 3.1. Patient Characteristics

Eighty patients were included in this study. The characteristics of the patients are shown in Table 1. 51 males and 29 females were included. The median age was 63 years (range 30–87 years). A history of prior cancer was found in 8 patients; 2 patients were formerly diagnosed with breast cancer, 1 with prostate cancer, 2 with bladder cancer, 1 T-lymphoblastic lymphoma, 1 with malignant melanoma, and one patient with testicular cancer. Ninety percent of the patients were considered in good performance status at the time of referral but 35 patients (44%) had concomitant diseases. The most common concomitant disease was hypertension (18%). Other concomitant diseases were diabetes, chronic obstructive respiratory disorders, or cardiovascular diseases.

### 3.2. Tumour Characteristics

The most common anatomic sites of primary tumours were the stomach 28 (35%) followed by the small intestine 21 (26%). Twenty one (26%) of the patients were considered as having local disease at the time of referral and 59 (74%) had metastatic disease. Forty five (55%) patients had metastasis to one organ, 10 patients (13%) to 2 organs, and 4 (5%) patients had metastasis to 3 or more organs. The liver was the predominant site for metastasis.

### 3.3. Treatment

#### 3.3.1. Response to Imatinib

All 80 patients received imatinib. Sixty nine (86%) of the patients were initially treated with 400 mg per day, while 10 (12%) patients were initially treated with 800 mg per day and 1 patient started with a reduced dose of 200 mg per day. Among patients initially treated with imatinib 400 mg daily 66 (85%) experienced clinical benefit (CR, PR, or SD) (Table 1). All patients starting with 800 mg had initially clinical benefit of the treatment but all had progressed at the time of evaluation.

At the time of evaluation 52 (65%) patients had progressed during imatinib treatment. Fifteen patients with local progression were treated with surgery or radio frequency ablation (RFA) to eliminate the resistant metastasis. The median OS was 21 months for patients who could not undergo local treatment (95% CI 4–39 months) compared to 54 months (95% CI 24–84 months) for patients who were suited for local treatment. 

Among the 35 patients increased in imatinib to a dose of 800 mg daily as a consequence of disease progression, 17 (49%) patients experienced clinical benefit (PR + SD).

The median TTP for patients initiated with 400 mg of imatinib was 23 months (95% confidence interval 11–38 months). The median TTP for patients initiated with 800 mg imatinib was 38 months (95% confidence interval 31–45 months). Among the patients increased in imatinib to a dose of 800 mg the median TTP was 3 months (range 2–4 months). At the time of evaluation 21 (26%) patients were alive and without progression. 

Using univariate analysis age, gender, performance status, concomitant disease, and different serum parameters such as haemoglobin, neutrophil count, lactate dehydrogenase level were not prognostic regarding TTP. Low serum sodium levels at the beginning of the treatment was associated with a significant shorter TTP compared to patients with normal serum sodium levels (*P* < 0.05). Low serum sodium was seen in 14 patients.

#### 3.3.2. Toxicities of Imatinib

Fourteen (20%) patients treated with imatinib 400 mg per day experience grade 3 or 4 toxicity which lead to permanent dose reduction. The toxicities seen were skin rash (33%) and gastrointestinal side effect (26%). Fatigue and facial oedema were seen but did not lead to dose reduction because the side effects were mild or transient. Among 45 patients who were treated with 800 mg of imatinib, 8 were reduced because of side effects. Overall 4 patients terminated the treatment as a consequence of side effects and 3 patients refused to continue treatment. Table 1 summarises the toxicity profile of imatinib treatment.

#### 3.3.3. Response to Sunitinib

Thirty two patients received sunitinib as 2nd-line treatment after verified radiological progression. Nineteen (60%) patients were initiated at a dose of 37.5 mg per oral daily without interruption, 10 (31%) patients were initiated at a dose of 50 mg daily 4 weeks while 3 (9%) patients were initiated with a reduced dose of 25 mg daily. Twenty six (82%) patients experienced clinical benefit (PR or SD). Three patients did not response to the treatment at any time, and 3 patients died before evaluation of response (Table 1). Four of 7 patients who did not show any response to imatinib had clinical benefit of sunitinib treatment. Five patients experienced local progression; among these, 2 were treated with surgery. At the time of evaluation 19 patients had progressed during the treatment.

The median TTP from the beginning of treatment with sunitinib was 9 months (95% confidence interval 5–13 months). At the time of evaluation 14 (44%) of the sunitinib-treated patients were still alive and 8 patients without evidence of progression.

#### 3.3.4. Toxicities of Sunitinib

Seven patients were reduced in dose as a consequence of side effects, primarily because of dermatological toxicity and musculoskeletal side effects (6 out of 7 patients). Treatment was terminated for 1 patient because of extensive mucosal toxicity and declining nutritional status. Two patients refused to continue treatment with sunitinib.

#### 3.3.5. Overall Survival

**Figure 1:**
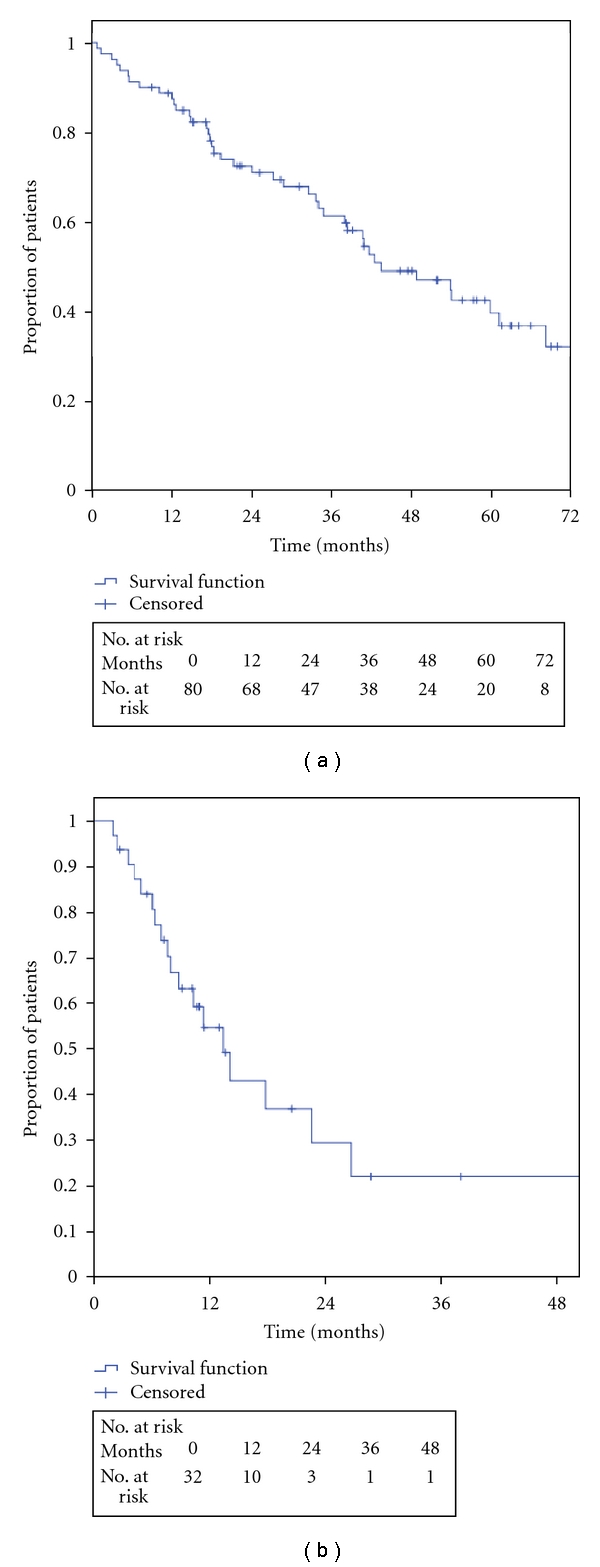
Overall survival after imatinib and sunitinib treatment.

**Figure 2:**
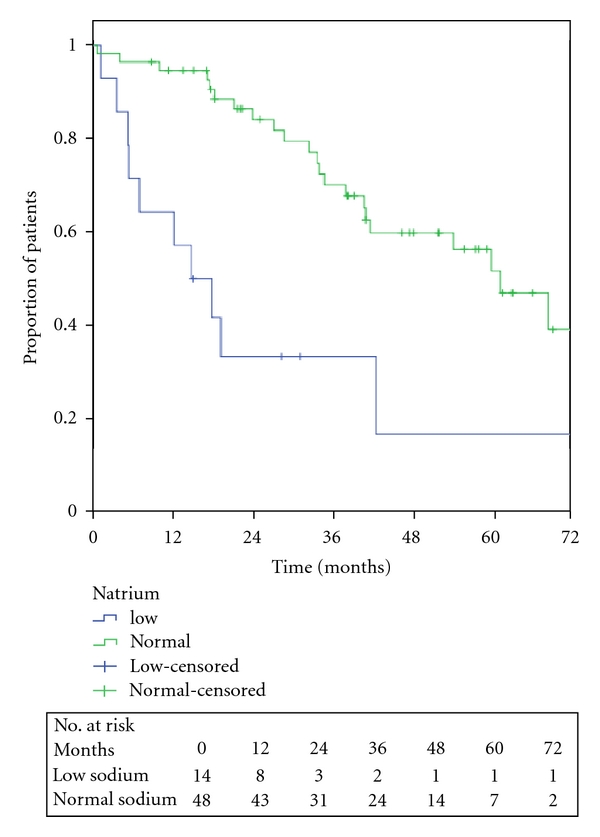
Relation between OS and high/low serum sodium.

The OS from the start of imatinib (a) and sunitinib (b) are shown in Figure 1, respectively. The 5-year OS for imatinib was 40% and sunitinib shows a 2-year OS of 30%. Gender, performance status, concomitant diseases, primary tumour site, LDH, haemoglobin, and neutrophil cell count were not found as prognostic factors (Table 2). Low serum sodium at the start of imatinib treatment was associated with reduced OS (Figure 2). Fourteen patients with low serum sodium at the beginning of imatinib treatment showed a median OS of 15 months (95% CI 5–25 months) compared to patient with normal serum sodium who had a median OS of 61 months (95% CI 47–76 months). The difference was statistically significant both in an unvariate analysis and in a multivariate model including haemoglobin and performance status (*P* < 0.05). Only when the serum sodium level was below normal range it affects OS. When dividing serum sodium into high and low level according to the mean value, no significant difference was seen.

## 4. Discussion

Before the use of imatinib the median OS of unresectable and metastatic GIST was 5–15 months [4, 13]. After the introduction of imatinib, a comprehensive meta-analysis including 1640 patients [8] described a median OS of 57 months. In this paper the median OS was 44 months (95% CI: 29–57 months). The difference between these results could be explained by the fact that this study is a retrospective clinical study without strict eligibility criteria such as concomitant disease and previous malignancies. The median age in our patients' cohort is slightly older than in other trials [8, 14, 15] which may affect OS. It is to be noted that the survival of the patients with normal serum sodium is 60 months that is comparable to those reported by randomised clinical trials.

Data from the literature describe median TTP during imatinib treatment in the range 18–37 months [6–8]. Our result shows a similar median TTP of 27 months (95% confidence interval 17–37 months). Clinical benefit (CR + PR and SD) has been reported in about 80% of the patients [6, 7, 14, 16] which also corresponds well with our findings with an initial clinical benefit rate of 83%. About 50% of the patients who progressed during treatment with imatinib 400 mg experienced clinical benefit after increasing the dose of imatinib as previously seen [15]. It is expected that about 11–14% of all patients will not benefit from imatinib treatment as seen in this study [14–17]. Thirteen patients (16%) experienced progression during imatinib 400 mg as best response; among these, 2 patients had clinical benefit when increasing the dose of imatinib. 

Surgical excision of local progression is recommended in order to eliminate resistant clones and in order to delay the change or modification of imatinib therapy. 

In general, imatinib was well tolerated. Twenty two % of the patients in this study experienced grade 3 or 4 toxicity; the most frequent toxicity seen was dermatological as expected [15, 17]. Facial oedema was almost always of low grade and diminished with time, which has also been shown previously [16].

Since 2005 Sunitinib has been used as 2nd-line treatment in the case of disease progression or intolerance to imatinib therapy [18]. The largest sunitinib study is a randomised double-blinded, placebo-controlled, multicentre international trial with 312 patients of which 207 received sunitinib. This study calculated a median TTP of about 6 months, and 65% of the patients had clinical benefit of the treatment [19]. Newer studies have shown a median TTP of 7–9 months and 53–80% of the patients experienced clinical benefit of the treatment. [20, 21]. In our study the median TTP was 9 months (95% confidence interval 3–13 months), and 82% of the patients experienced clinical benefit of the treatment. The patients in this study were a little older, but more patients had performance status 0 when compared to the randomised trial. Twenty two % of the patient experienced toxicity compared to 40% in the randomised trial. 

This study pointed toward low serum natrium as a poor prognostic factor regarding TTP as well as OS. This observation could not be explained by concomitant diseases among the patients with low sodium nor could the performance status at the start of imatinib treatment. All except one patient who showed a reduced level of serum sodium had a normal creatinine level, and there were no difference in patients' characteristics between the groups with low and high sodium (data not shown). No other haematological or electrolytic parameters were found to be significant regarding their prognostic value. 

Hyponatremia is a common phenomenon in cancer patients and often associated with poor prognosis. The prognostic value of serum sodium has been investigated for small-cell lung cancer [22], metastatic renal cell carcinoma [23], hepatocellular carcinoma [24], and gastric cancer [25]. In the mentioned studies low serum sodium was found to reduce OS. Furthermore, low serum sodium at the time of hospitalisation regardless of the cause is associated with high mortality [26]. Even though this study only includes 14 patients with low serum sodium, hyponatremia significantly reduced the OS compared to patients with normal serum sodium at the time of initiating treatment. None of our patients had other causes of hyponatremia such as heart failure, cirrosis, or nephritic syndrome, and all except one patient with low serum sodium had normal creatinine indicating normal renal function. This was confirmed by a multivariate analysis including haemoglobin, performance status and serum sodium.

Other studies have showed the following adverse prognostic factors: high grade primary tumours, poor performance status, high neutrophil counts, low haemoglobin level, male sex, and GIST from small bowel origin [8, 10]. None of these factors influenced the OS significantly in this study. The prognostic value of these factors has been tested regarding time to progression. Because patients in this study were treated with different doses of imatinib and increased or local treatment was initiated if radiological progression was observed, we did not look at prognostic factor regarding initial or late resistance to imatinib. One study has looked for prognostic factors for overall survival and found that poorer performance status, male sex, high absolute neutrophil count, and low albumin were significantly associated with worse overall survival [6]. A good performance status was in this study defined as PS 0 whereas the study by Blake tested PS 0 or 1 versus PS 2 or 3. The number of patients in this study in performance status 2 or more was 8 patients and no significant effect was observed using PS 0 and 1 versus PS 2 and 3. By testing absolute neutrophil count and by categorising the count as normal and high, we found no significant (*P* = 0.291) decrease in OS presumably because of the low number for patients in this study.

In summary, these data from a single institution confirm that by following the general recommendations, the overall excellent clinical outcomes achieved with the use of TKIs in advanced GIST are as would be expected from clinical trials. The data pointed toward low serum sodium at the start of imatinib treatment as an adverse prognostic factor. However, this statement needs to be confirmed in lager studies including more patients.

## Figures and Tables

**Table 1:** Patient and treatment characteristics.

	Imatinib *n* = 80 (%)	Sunitinib *n* = 32 (%)
Sex		
Male	51 (64)	10 (31)
Female	29 (36)	22 (69)

Age, years		
Median (years)	63	61
Range	30–87	40–85

Performance status		
0	54 (68)	24 (75)
1	18 (22)	6 (19)
2	7 (9)	1 (3)
3	1 (1)	1 (3)

Concomitant disease		
No	45 (56)	21 (66)
Yes	35 (44)	11 (34)

Prior malignancy		
No	72 (90)	28 (88)
Yes	8 (10)	4 (12)

Primary treatment		
Surgery	49 (61)	22 (69)
No prior surgery	31 (39)	10 (31)

Start dose of imatinib/sunitinib		
800 mg/50 mg	10 (13)	10 (31)
400 mg/37,5 mg	69 (86)	19 (60)
200 mg/25 mg	1 (1)	3 (9)

Permanent reduction of start dose		
No	61 (76)	25 (78)
Yes	19 (24)	7 (22)

Reason for reduction of start dose		
Haematological	2 (11)	
Musculoskeletal	2 (11)	3 (43)
Multisystem	1 (5)	
Dermatological	7 (37)	3 (43)
Gastrointestinal	6 (31)	
Other	1 (5)	1 (14)

Response^(a)^		
CR	3 (4)	
PR	47 (59)	13 (41)
SD	16 (20)	13 (41)
PD	13 (16)	3 (9)
Death before evaluation	1 (1)	3 (9)

Progression^(b)^		
No	26 (33)	8 (25)
Yes	52 (65)	19 (59)
Not evaluable	2 (2)	5 (16)

Local treatment		
No	40 (77)	17 (89)
Yes	12 (23)	2 (11)

^(a)^Best response at any time during treatment. CR: complete response, PR: partial response, SD: stable disease, PD: progression of disease.

^(b)^Progression at the time of evaluation.

**Table 2:** Univariate and multivariate analysis of potential prognostic factors affecting overall survival.

	Univariate analysis			Multivariate model		
	No. of patients	No. of events	*P*	No. of patients	HR	*P*
Gender						
Male	51	26				
Female	29	16	0.453			

PS						
0	54	26		45	1	
1–3	26	16	0.207	24	1.2	0.627

Concomitant disease						
No	45	23				
Yes	32	19	0.353			

Site of primary tumour						
Small intestine	21	10				
Other	59	32	0.213			

Serum sodium						
Normal	55	22		55	1	
Low	14	11	**<0.01**	14	0.3	**0.04**

Serum LDH						
Normal	44	21				
Low	29	16	0.846			

Serum Hb						
Normal	21	8		20	1	
Low	52	29	0.099	49	0.7	0.285

Neutrophil count						
Normal	57	29				
High	16	8	0.745			
